# The Role of Panendoscopy in Primary Diagnostics of Patients with Oral Cavity Cancer

**DOI:** 10.1007/s12663-024-02363-6

**Published:** 2024-11-26

**Authors:** Katherina Jordan, Lucas Ritschl, Andreas Fichter, Klaus Dietrich Wolff, Lukas Greber, Markus Nieberler

**Affiliations:** 1 Department of Oral and Maxillofacial Surgery, University Hospital Rechts der Isar, Technische Universität München, Ismaninger Straße 22, 81675 Munich, Germany; 2 Department of Oral and Maxillofacial Surgery, Ludwig Maximilian University of Munich, Lindwurmstraße 2a, 80337 Munich, Germany

**Keywords:** Head and neck cancer, Oral cavity cancer, OSCC, Oral squamous cell carcinoma, Cancer in head and neck region, Synchronous malignancy, Second primary, Synchronous second cancer, Synchronous neoplasms, Simultaneous second primary carcinoma, Multiple primary cancers, Staging, Endoscopy, Triple endoscopy, Panendoscopy, Staging

## Abstract

**Purpose:**

Panendoscopy is known as a standard procedure in the staging of oral cavity cancer (OCC), which is intended to rule out synchronous second carcinomas (SSC) (Metzger K et al in J Craniomaxillofac Surg 47(12):1968–1972, 2019; Priante et al. in Curr Oncol Rep 13(2):132–137, 2011; Stoeckli et al. in Otolaryngol Head Neck Surg 124(2):208–212, 2001; Sharma et al in Laryngorhinootologie 92(3):166–169, 2013). However, the value in relation to the detection of SSC in the upper aerodigestive tract is questionable (Deutsche Gesellschaft für Mund- Kiefer- und Gesichtschirurgie (DGMKG), S3-Leitlinie Diagnostik und Therapie des Mundhöhlenkarzinoms. https://www.leitlinienprogramm-onkologie.de/leitlinien/mundhoehlenkarzinom/, 2021). The aim of the study was to redefine the role of panendoscopy in the staging of OCC—not only with regard to the detection of SSC—as the arrangement of panendoscopy is subject to further influencing factors. In addition, the diagnostic added value and effects on the therapy of the index tumor were elicited.

**Material and methods:**

A retrospective review of 191 patients with a confirmed diagnosis of OCC was conducted, between January 2018 and December 2019, at the Department of Oral and Maxillofacial Surgery of the clinic of the Technical University of Munich, Germany. Panendoscopy included inspection and palpation of the oral cavity and oropharynx, epipharyngoscopy, microlaryngoscopy, and rigid esophagoscopy.

**Results:**

The following parameters had a statistically significant influence on the decision to perform panendoscopy in primary diagnostics: risk factors, ENT status, and imaging. Panendoscopy was indicated in the primary diagnostics due to a suspicion of an SSC in 22.5% of patients and due to recurrence in 29%. The exact determination of localization and assessment of tumor extent was the decisive indicator for panendoscopy in 25.8% of patients. Of the 31 panendoscopies performed, a tissue sample was obtained in 67.7% (n = 21); none of the suspected cases proved to be an SSC.

**Conclusion:**

Panendoscopy in the primary diagnostics of OCC should not be routinely indicated (Koerdt et al in Anticancer Res 41(4):2039–2044, 2021), but should be indicated on an as-needed basis, taking patient-specific criteria into account. In addition to ENT status and imaging, the risk factors of smoking and alcohol should be considered. In patients with unremarkable mirror and radiological findings and no risk factors, panendoscopy can be omitted without further risk (Metzger K et al in J Craniomaxillofac Surg 47(12):1968–1972, 2019; Koerdt et al in Anticancer Res 41(4):2039–2044, 2021).

## Introduction

Panendoscopy in the staging of oral cavity cancer (OCC) (95% oral squamous cell carcinoma [1–3]) is known as a standard procedure, which should serve to exclude synchronous second carcinomas (SSC) [4–7]. In the German S3 guideline [8] the decision to perform a panendoscopy refers to the presence of abnormalities in the mirror examination routinely performed by the otorhinolaryngologist and/or in the imaging (CT/MRI) [8]. However, the value of panendoscopy in detecting SSC in the upper aerodigestive tract, especially in the pharynx or larynx [9–12], is questionable [8].

Only a minority of SSC are detected by endoscopy alone [6, 13, 14]. According to recent studies, the detection rate of SSC by panendoscopy in the primary diagnostics of OCC is about 1.1% [13, 15, 16]. In the absence of risk factors in the medical history, the percentage is down to 0% [17].

The aim of the study was to review the value of panendoscopy for the detection of SSC and to redefine its role in the staging of OCC, as the arrangement of panendoscopy is subject to other influencing factors. The added diagnostic value as well as implications for therapy were examined.

## Material and Methods

A retrospective review was conducted of 191 patients with a confirmed diagnosis of OCC, between January 2018 and December 2019, at the Department of Oral and Maxillofacial Surgery of the clinic of the Technical University of Munich, Germany. Panendoscopy included inspection and palpation of the oral cavity and oropharynx, epipharyngoscopy, microlaryngoscopy, and rigid esophagoscopy. The detection of SSC was topographically limited to the upper aerodigestive tract (pharynx, larynx) and esophagus. SSC was defined as such carcinoma diagnosed at the same time as the index tumor or within six months [4, 18, 19].

### Data Collection

Data were generated from the hospital’s internal SAP patient management system (SAP, Walldorf, Germany) using the classification according to ICD-10-GM (International Statistical Classification of Diseases and Related Health Problems, German Modification [20]). The search was limited to patients with inpatient stays in the Department of Oral and Maxillofacial Surgery. To investigate in which cases panendoscopy was performed in the primary diagnostics, the following variables were considered: ASA class, tumor location and entity, UICC stage, risk factors, ENT status, and imaging.

### Risk Factors

Patients were considered smokers if they smoked regularly. Alcohol consumption was defined according to specific thresholds (women > 12 g per day, men > 24 g per day). Previous tobacco and/or alcohol use was considered as positive. Patients without risk factors were those who had never been exposed to tobacco or alcohol use.

### ENT Status and Imaging

**Fig. 1:**
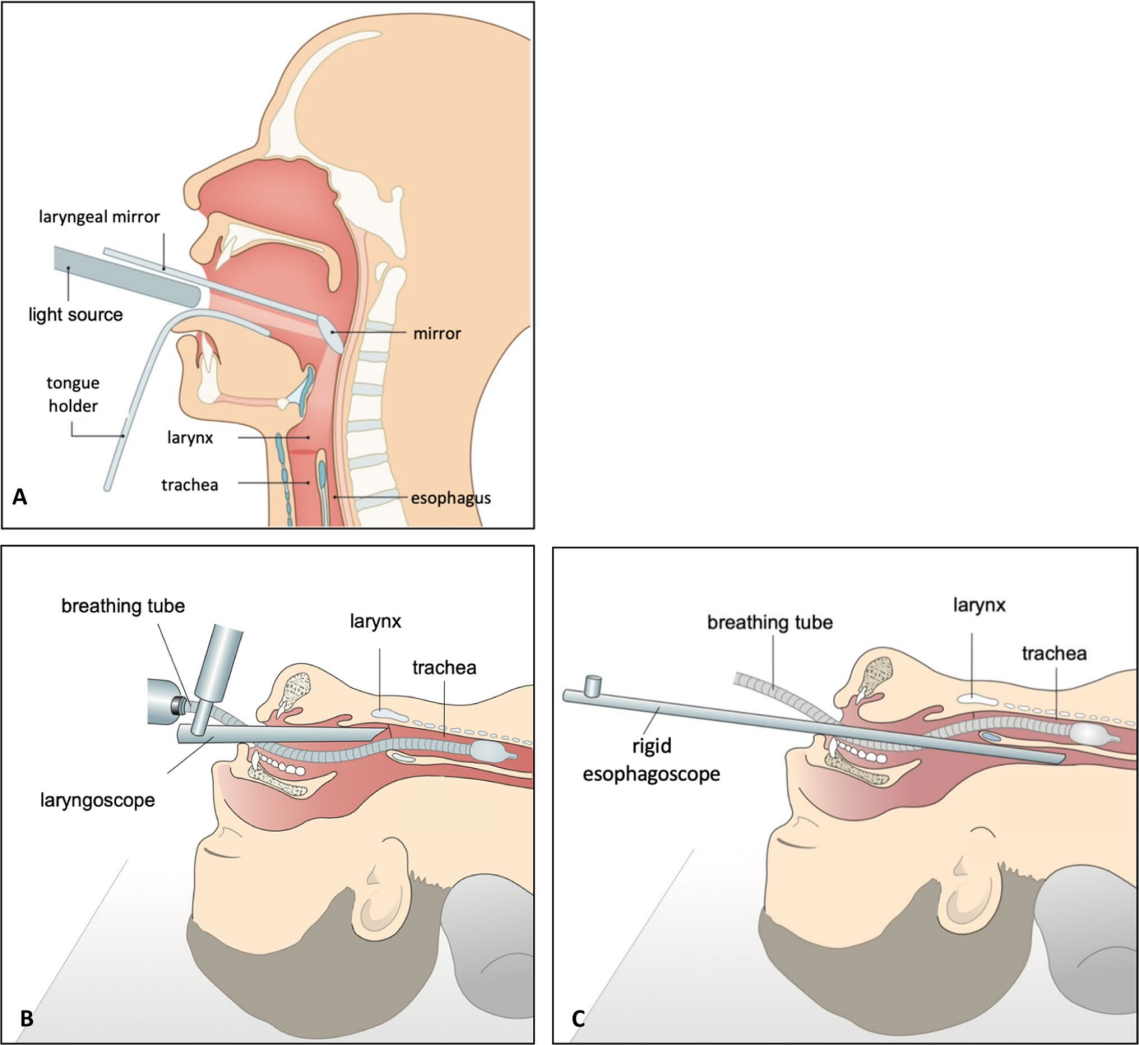
Schematic representation of mirror examination (ENT status) and panendoscopy. **A** Schematic representation of a diagnostic mirror examination (ENT status). According to Zacharias: Look into the throat [42]. **B** Microlaryngoscopy. **C** Rigid esophagoscopy. From the information sheet HNOE06, Thieme Compliance System [43]

If clinical abnormalities regarding an SSC in the pharynx (oropharynx and hypopharynx) and/or larynx were documented in the consultation report, the ENT status was considered positive (Fig. 1). Abnormalities in the imaging (CT/MRI) with suspicions of an SSC were also considered positive.

The indication and timing of panendoscopy were relevant to the study. To determine the diagnostic added value of panendoscopy and the therapeutic effects, the sample collections in panendoscopy were analyzed.

## Results

Out of 191 cases with OCC, a panendoscopy was performed in 31 cases in the primary diagnostics. The following parameters significantly influenced the decision whether panendoscopy was performed: the existence of risk factors, and abnormal findings in the ENT status or in the imaging. Examination of the other parameters (ASA class, tumor location and entity, UICC stage) did not yield statistically valid results (Table 1).

**Table 1:** Overview of the 191 cases and influencing parameters in primary diagnostics in % (n)

Performance of a panendoscopy in the 191 cases			
In after-care 3.7% (7)	Alio loco 1.6% (3)	No panendoscopy performed 78.5% (150)	In primary diagnostics 16.2% (31)
*Risk factors*			
Alcohol			
Positive		73.7% (56)	26.3% (20)
Negative		89.5% (94)	10.5% (11)
Smoking			
Positive		73.4% (69)	26.6% (25)
Negative		93.1% (81)	6.9% (6)
*ENT status*			
Positive		40.0% (2)	60.0% (3)
Negative		88.1% (141)	11.9% (19)
Limited assessable		43.8% (7)	56.2% (9)
*Imaging (CT/MRI) with suspicions of an SSC*			
Positive		60.0% (9)	40.0% (6)
Negative		84.7% (127)	15.3% (23)
Unknown		87.5% (14)	12.5% (2)
*Tumor locations*			
Tongue edge		75.0% (36)	25.0% (12)
Mandible		96.9% (31)	3.1% (1)
Anterior floor of the mouth		80.0% (16)	20.0% (4)
Cheek mucosa		93.3% (14)	6.7% (1)
Lateral floor of the mouth		80.0% (8)	20.0% (2)
Maxilla Base of the tongue		90.0% (9) 50.0% (2)	10.0% (1) 50.0% (2)
Other*		80.1% (34)	19.9% (8)

*Palate, lips, other locations of the tongue and of the floor of the mouth, multifocal

### Parameters Influencing the Decision to Perform Panendoscopy in Primary Diagnostics (Fig. 2)

**Fig. 2:**
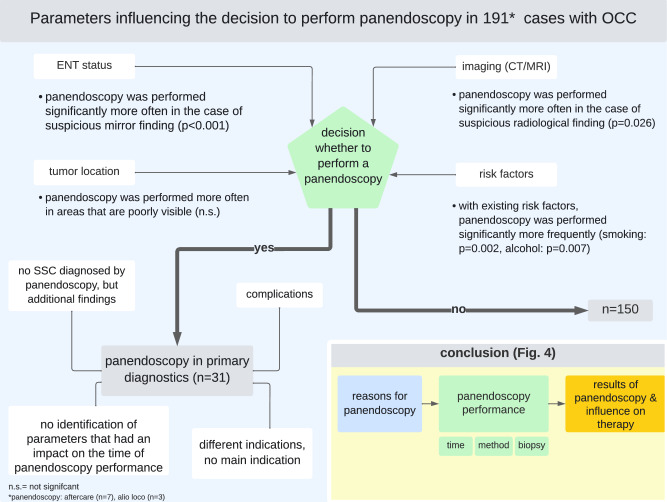
Subanalysis to consider the additional effect of alcohol in the smoking and no-smoking groups in A, or the additional effect of smoking in the alcohol and no-alcohol groups in B

#### Tumor Entity and Location of the Index Tumor

With 115 cases, squamous cell carcinomas accounted for the majority of all entities, followed by recurrences of squamous cell carcinomas (39). Other carcinomas such as dermatofibrosarcoma were comparatively rare. Endoscopic examination was not performed in these rare cases.

In principle, OCC can occur anywhere in the oral cavity, but some regions, such as the tongue or floor of the mouth, are commonly affected [21–23]. The most common location of OCC was tongue edge (n = 48). In relative terms, it can be concluded that panendoscopy was performed more frequently for tumors in areas that are poorly visible, such as the base of the tongue.

#### Risk Factors

The significant influence of the risk factors of smoking and alcohol on the decision to perform a panendoscopy in the primary diagnostics could be demonstrated. If a patient reported alcohol consumption in the medical history, panendoscopy was performed significantly more often (*p* = 0.007). Panendoscopies were performed more than three times as often among patients who reported smoking as a risk factor (*p* = 0.002).

**Fig. 3:**
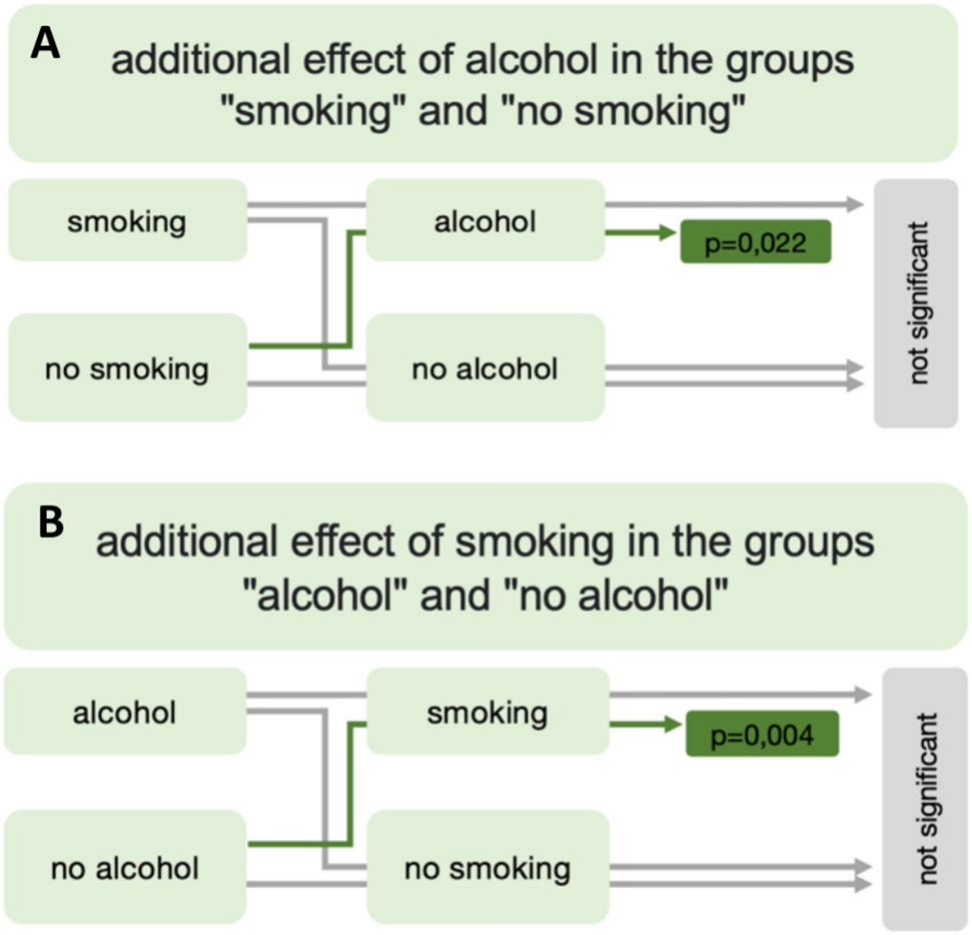
Schematic representation of the results

In a subanalysis, the question was considered to what extent the risk factor alcohol within the smoking and no-smoking groups additionally influenced the likelihood of a panendoscopy being performed (Fig. 3). The additional effect of alcohol could only be detected in the group of patients without the risk factor smoking (*p* = 0.022). The result was analogous to the question about the additional effect of the risk factor smoking.

#### ENT Status and Imaging

In cases of positive (60%) and limited assessable (56.2%) ENT status, panendoscopy was performed significantly more often (*p* < 0.001) than in cases of unremarkable mirror findings (11.9%).

Similarly, when imaging was suspicious, panendoscopy was performed significantly more often (*p* = 0.026).

### Examination of the 31 Panendoscopy Cases

#### Indications for Panendoscopy

Retrospectively, no main indication for panendoscopy could be identified. Panendoscopy in primary diagnostics was not only indicated due to a suspicion of an SSC (22.5%); recurrence situations (29%) or the exact localization and extent of the index tumor (25.8%) were also indicators.

#### Timing of the Panendoscopy

In patients with OCC with surgical therapy, panendoscopy can be performed before surgery (preoperative) or simultaneously as part of surgery (intraoperative). Before surgery means that the patient must undergo a separate general endotracheal anesthesia (GETA) session as part of primary diagnostics. In patients with non-surgical measures (radio-, chemo-, radiochemo-, immunotherapy or best supportive care), a GETA must always be scheduled for panendoscopy. Overall, there was no identification of parameters that had a statistically significant effect on timing. Relative consideration of patients treated surgically yielded the following results: In patients with recurrence, panendoscopy was most commonly performed preoperatively (80%). In patients with unclear tumor extent and location, panendoscopy was more frequently (57%) performed at the same time as surgery.

#### Biopsy in Panendoscopy

Of the 31 panendoscopies performed in primary diagnostics, a tissue sample was obtained in 67.7% (n = 21). There was no case in which an SSC could be diagnosed. Additional findings included cysts (e.g., vallecular cyst, hypopharyngeal cyst, epiglottis cyst), leukoplakia (localized on the palate, vocal folds, apical tonsillar lodge), and florid inflammatory signs. The suspicion of an SSC arose in advance clinically by ENT status and/or radiologically (19%, n = 4) or only during panendoscopy (9.5%, n = 2). The most decisive reason for a biopsy was to determine the location and extent of the index tumor, with 47.6% (n = 10). However, when all 191 cases were considered, three SSC were diagnosed in the oral cavity which had not yet appeared visibly (clinically, radiologically, and endoscopically) at the time of primary diagnostics.

#### Performance of Rigid Esophagoscopy

Rigid esophagoscopy was performed in more than half (58.1%) of all cases. Reasons for discontinuation (6.5%) were upper esophageal sphincter narrowing or nonmobilization of the cervical spine (e.g., plate osteosynthesis in the cervical spine). Reasons why rigid esophagoscopy was not performed as part of panendoscopy (12.9%) were postradiogenic esophageal stenosis, severely restricted mouth opening, or already performed panendoscopy.

## Discussion

### The Role of Panendoscopy in the Primary Diagnostics of OCC

**Fig. 4:**
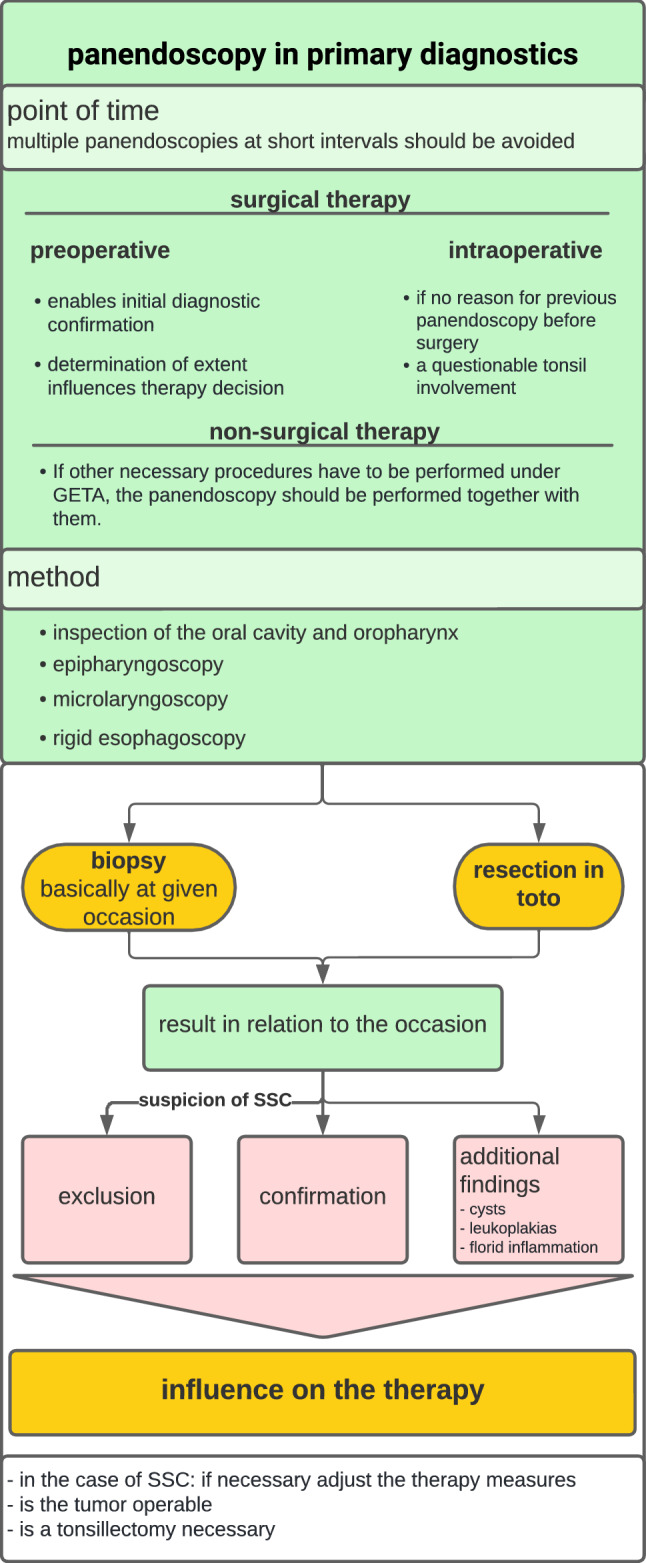
Panendoscopy in the primary diagnostics of OCC

It is obvious that panendoscopy can be helpful not only regarding the possible detection of SSC but also in unclear tumor extent and recurrence situations in primary diagnostics (Fig. 4). If it is not feasible to confirm the diagnosis of the index tumor by sampling under local anesthesia, a biopsy is made possible during panendoscopy. If the ENT status reveals abnormalities in the oropharynx that can still be viewed and biopsied, a histological examination can be performed directly by taking a sample under local anesthesia. Biopsies in deeper areas of the pharynx, larynx, and esophagus are only possible by endoscopic procedures under GETA. Sometimes patient-specific criteria such as incompliance, difficult mouth opening, lockjaw, gag reflex, and copious salivation are added, making accurate evaluation and biopsy in mirror examination (ENT status) impossible.

In particular, in recurrent situations, there are often difficult conditions for mirror examination. Anamnestically recurrent patients have already undergone a form of therapy: depending on radiation or surgery, postradiogenic swelling, dry or slimy conditions of the mucous membranes, and flap reconstruction may hinder insight. Under such conditions, an SSC cannot be excluded with certainty. In this case, however, it is not the exclusion of an SSC that indicates panendoscopy; rather, it is the recurrence situation (see above) itself that makes it impossible to assess the extent of the tumor or to biopsy the site suspected of recurrence under local anesthesia.

Samples taken during panendoscopy not only histologically clarify the suspicion of an SSC—if SSC is excluded, additional findings such as cysts, florid inflammation, or leukoplakia can be diagnosed at the same time. An additional advantage is the possibility of direct resection of these during panendoscopy, so that no more therapeutic measures are required.

Another value of panendoscopy as a diagnostic tool is that, in contrast to tomography, it also allows the detection of inflammatory and dysplastic changes, including those that are often shallow in their growth [4, 24]. Further, artifacts due to dental prostheses are frequent in imaging (in this study in 39.8% of the 191 cases) and limit precise diagnosis.

Regarding the importance of panendoscopy in primary diagnostics, the crucial question is to what extent findings from panendoscopy have an impact on therapy for the index tumor. In this study, no SSC was diagnosed in any panendoscopy during primary diagnostics, so no therapy adjustment for the index tumor had to be made. Nevertheless, it should be said: If an SSC is detected, for example in the esophagus, it may well be that changes in the therapy concept have to be made [25, 26]. Panendoscopy had a major influence on therapy when the extent of a tumor – which could only be determined by panendoscopy – was the basis for deciding whether resection was still justifiable. This may occur in tumorous processes with multifocal extension. A questionable involvement of the tonsils can also be clarified by sampling in panendoscopy. Indications of such involvement may be conspicuous asymmetries in the imaging, whereby it is always important to consider the patient’s case with their history: Even a previous tonsillectomy can lead to such an appearance and must not be misinterpreted. In the case of malignant involvement of the tonsils, this finding has an impact on the extent of therapy in which tumor resection includes tonsillectomy.

### The Value of Panendoscopy Regarding the Detection of SSC

Incidence data on SSC are low in recent studies (1–4.8%) [6, 27–32]. The knowledge that risk factors (especially smoking and alcohol) promote malignancies is well known. Risk factors are closely related to the concept of field cancerization, which explains the etiopathogenesis of SSC [4, 33]. Consistent with the results of this study, recent studies have shown an increasing trend toward risk stratification when panendoscopy is performed in the primary diagnostics: Patients without risk factors have a low risk of developing an SSC in the upper aerodigestive tract. Thus, in terms of the ability to detect SSC, patients without risk factors do not benefit from panendoscopy during staging [4, 13, 15, 34, 35].

Of the 31 cases with panendoscopy in the primary diagnostics of this study, there was no case in which an SSC could be diagnosed. However, when all 191 cases were considered, three SSCs were diagnosed in the oral cavity. This result questions the value of panendoscopy for the detection of SSC. These three tumors had not yet appeared visibly (clinically, radiologically, and endoscopically) at the time of primary diagnostics of the index tumor.

The definition of an SSC is variable and has been described differently in studies [4, 6, 13, 18, 19, 36]. The definition of “synchronous” does not exclude a temporally delayed visible occurrence. Thus, the timing of diagnosis of SSC should be viewed critically, because most carcinomas are initially clinically asymptomatic, and the patient groups that are affected often ignore presenting symptoms [6]. In cases, where SSC occurred with a time delay, panendoscopy does not provide any benefit in the context of primary diagnostics; here, retrospectively a panendoscopy would have been better indicated at a later time point. In view of the tumor location of these three SSC (all three located in the oral cavity), panendoscopy would not have provided any added value. Recent studies postulate that the SSC of an OCC are primarily located in the pharyngeal and laryngeal regions [13, 18, 37]. Gaining knowledge of the interrelationships of tumor locations in the occurrence of SSC should be considered in the scope of the diagnostic procedure [18]. Thus, the question arises whether rigid esophagoscopy should be part of every panendoscopy.

In addition to indisputable microlaryngoscopy, routine esophagoscopy is advocated as part of panendoscopy according to the esophageal cancer guideline [18, 26]. Prospective studies showed that endoscopic screening allows early detection of SSC in the esophagus and curative therapy [25, 26]. The main advantage of endoscopy with rigid tubes is that the contracted portion of the upper digestive tract, especially the esophageal inlet and also the hypopharynx, can be better unfolded and stretched open, so that even minor malignant changes in this area are not missed [18, 38]. Some studies report a higher risk of perforation when using rigid sheaths [6, 13]. Worth mentioning is that rigid esophagoscopy is not always feasible or may have to be discontinued (see results section).

There is also critical discussion of delaying the start of tumor therapy by prior panendoscopy [4, 8, 39, 40]. In general, OCC progress more rapidly than other cancers, and it is this that must be considered when prioritizing the course of treatment [41].

## Conclusion

Panendoscopy in the primary diagnostics of OCC should not be performed routinely [15], but should be indicated based on patient-specific criteria. The basis for this is a detailed and accurately documented medical history. In addition to ENT status and imaging, risk factors should be considered. Nevertheless, even endoscopic examination does not provide diagnostic certainty [18]. In patients with unremarkable mirror and radiological findings and no risk factors, panendoscopy to exclude SSC can be omitted [4, 15]. In order to conclusively evaluate the value of panendoscopy in primary diagnostics for the detection of SSC, further knowledge is needed with regard to etiopathogenesis, especially temporal occurrence and location in connection with the principle of field cancerization.
